# A Systematic Review of the Influence of Bovine Colostrum Supplementation on Leaky Gut Syndrome in Athletes: Diagnostic Biomarkers and Future Directions

**DOI:** 10.3390/nu14122512

**Published:** 2022-06-17

**Authors:** Hanna Dziewiecka, Harpal S. Buttar, Anna Kasperska, Joanna Ostapiuk-Karolczuk, Małgorzata Domagalska, Justyna Cichoń, Anna Skarpańska-Stejnborn

**Affiliations:** 1 Department of Biological Sciences, Faculty of Physical Culture in Gorzów Wielkopolski, Poznan University of Physical Education, Estkowskiego 13, 66-400 Gorzów Wielkopolski, Poland; annakasperska.awf@gmail.com (A.K.); joanna.ostapiuk@gmail.com (J.O.-K.); m.domaglaska@icloud.com (M.D.); cichonjustyna95@gmail.com (J.C.); ankass@poczta.onet.pl (A.S.-S.); 2 Department of Pathology & Laboratory Medicine, Faculty of Medicine, University of Ottawa, Ottawa, ONT K1H 8M5, Canada; hsbuttar@bell.net

**Keywords:** bovine colostrum, gut permeability, I-Fabp, athletes, dual sugar test, exercise, zonulin

## Abstract

Background: Bovine colostrum (BC) contains a myriad of bioactive molecules that are renowned for possessing unique medicinal benefits in children and adults, and BC supplements are considered safe and cost-effective options to manage/prevent the incidence of upper respiratory tract infections and gut-related problems in athletes. In this review, we will try to answer the question: How will BC supplementation ameliorate gut permeability problems among athletes? Methods: Literature searches were performed using PRISMA guidance to identify studies assessing the influence of BC supplements on gut permeability. Studies were selected using four databases: PubMed, Web of Science, Scopus, and EBSCO, and a total number of 60 articles were retrieved by using appropriate keywords. Results: Nine studies were selected that met the eligibility criteria for this review. The data analysis revealed that vigorous exercise profoundly increases intestinal permeability, and BC supplementation helps to reverse gut permeability in athletes. Conclusion: BC supplementation may be highly beneficial in improving gut permeability in athletes. However, well-designed, placebo-controlled, and randomized studies are needed to evaluate the long-term safety and efficacy and to determine the optimal dose schedules of BC supplementation in high-performance athletes.

## 1. Introduction

Immune depression in athletes worsens through increasing training loads, psychological stress, disturbed sleep, and environmental extremes, which can contribute to an increased risk of respiratory tract infections [1]. Moreover, highly trained athletes are often exposed to crowds, foreign travel, poor hygiene, and training or competition venues that elevate their exposure to pathogens, leading to increased infections. Prolonged exercise, especially in excessively hot environment conditions, has shown to cause increased gut permeability, which may result in systemic toxemia [1]. The intestinal hyperpermeability or leaky gut syndrome has been reported as a pathological disorder linked to the loosening of tight junctions in the gut epithelial wall and to a reduction of the intestinal barrier’s protective integrity, thereby allowing the seepage of endotoxins from the lumen to leak into the bloodstream and/or other body organs [2]. de Oliveira et al. (2014) suggested that physical exercise may also disturb the immune system of the digestive tract and cause damage to the enterocytes, consequently resulting in increased inflammatory response and gastrointestinal disorders [3].

Furthermore, intensive exercise may negatively impact the digestive system and cause symptoms such as abdominal pain, colic, flatulence, nausea, vomiting, or diarrhea, which affect nearly 70% of athletes and occur 1.5–3 times more often among high-performance athletes than among amateurs [4]. Researchers have estimated that approx. 70% of the immune system is located in the gastrointestinal tract. Any type of intestinal ischemia is one of the important pathological factors that can cause cell damage in an athlete’s gut, negatively influence the immune function, and trigger an inflammatory response in the gut, thereby affecting the athletes’ health and performance [1]. The latest research results have shown that upper respiratory tract illness is the most common reason for non-injury-related presentation seen in sports medicine clinics (accounting for 35–65% of illness presentations), and some problems may be associated with gastrointestinal hyperpermeability and seepage of endotoxins in the systemic circulation [5]. Ischemia-induced damage in the gut also adversely affects the Paneth cells that secrete antimicrobial peptides and proteins, mucus-producing cells, and tight-junction proteins (e.g., claudin-2) that prevent pathogenic organisms from entering the bloodstream [1,4]. The intestinal sealing capacity of BC has also been attributed to BC-mediated induction of the intestinal barrier-strengthening cytokine TGF-β. Due to increased gut permeability, lipopolysaccharide (LPS) and pro-inflammatory cytokines can easily pass through the enteric cell lining [1,4]. 

According to recent reports, not only the value of VO_2_ max alone but also the exercise load performed above the threshold of anaerobic changes may constitute an important factor in causing damage to the gastrointestinal tract resulting from vigorous exercise [6]. In addition, it turns out that even heavy-duty exercises (e.g., fast running or relay race) lasting less than 10 min can markedly enhance the intestinal permeability [7], thus suggesting changes to the existing data indicating that the effort should last at least 2 h [8]. The results of recent reports reveal that the problems of intestinal hyperpermeability may occur not only in a broad group of athletes but also in physically active people. Acquiring science-based knowledge about the cause-and-effect relationships between the sealing of the intestinal barrier with the use of dietary supplements like BC and post-workout regeneration would be a key element in improving the health conditions of athletes, which would undoubtedly impact health and efficiency among athletes and physically active people. BC contains a wide array of anti-microbial and immune-boosting immunoglobulins, as well as anti-inflammation and growth-promoting factors that can contribute to the enhancement of the acquired and innate immune systems, including a range of peptides and proteins with direct antimicrobial effects [9]. 

Data extracted from the literature suggest that BC may have direct antimicrobial and endotoxin-neutralizing effects throughout the alimentary tract and other bioactivities that suppress gut inflammation and promote mucosal integrity and tissue repair under various conditions related to tissue injury. The BC constituents may also contribute to curing bowel inflammatory events (e.g., inflammatory bowel disease, IBD), resulting from localized anti-inflammatory effects after contact with the gut mucosa [9]. 

The BC components have been classified into (a) immunological factors, namely, immunoglobins, lactoferrin, lysozyme lactoperoxidase, microRNA, glycoconjugates, B and T lymphocytes, leukocytes, interleukins, and other polypeptide-rich prolines; (b) growth factors; and (c) nutrients. Apart from lactose, the level of the bioactive ingredients like fat, protein, peptides, minerals and vitamins, growth factors, cytokines, hormones, nucleotides in BC are the highest immediately after delivery and then begin to decline [2,10], most intensely within 72 h postpartum [10]. Many factors influence the quality of BC supplements, such as calving interval, breed, age, seasons, genetic factors, heat stress, and humidity [2], in addition to the technological processes and hygiene conditions of production [2,11]. Hałasa et al. [11] reported that the colostrum harvested two hours after delivery had the most substantial influence on gut permeability.

Another important constituent of colostrum is lactoferrin, which is an iron-binding multifunctional glycoprotein belonging to the transferrin family. It is present in most milk secretions and reaches particularly high concentrations in colostrum and breast milk [12]. It directly influences the growth and proliferation of enterocytes [12] and also has a strong antimicrobial activity [13]. Additionally, lactoferrin has an impact on the levels of cytokines and chemokines that are produced by GALT cells (gut-associated lymphoid tissue) [14] and creates an environment for the growth of beneficial bacteria in the gut [15]. It exerts a potential influence on the integrity of the gut barrier and prevents leaky gut syndrome. 

Immunoglobulins are the most crucial protein fraction of BC and are also produced by white blood cells in our bodies. A recent systematic review and meta-analysis analyzed the immunological effects of BC supplementation in trained and active individuals. The results of these studies showed that supplementation with BC had little or no effect on changes in serum immunoglobulin levels (IgA and IgG), as well as on levels of lymphocytes, neutrophils, and immunoglobulins (IgA) present in saliva [16]. This ineffectiveness was even more severe given that the doses used in different studies were 10–25 g/day, while commercial suppliers often recommend a daily intake of between 500 mg and 1 g/day [17].

BC contains insulin growth factor-I (IGF-1) that stimulates the growth and reconstruction of cells and tissues [18]. Animal studies suggest that IGF-I plays a key role in rebuilding the intestinal epithelium’s integrity after damage [19]. However, neonatal IGF-1 supplementation showed no differences between the placebo and the BC group in sugar tests after 14 and 21 days of supplementation [20]. In contrast, Mero and colleagues [21,22] reported significant increases in serum IGF-1 concentrations following BC supplementation for 14 days when examining athletes. Other researchers, who used similar doses during the same/or longer supplementation periods reported no changes. Other investigators including Kuipers et al. [23], Buckley et al. [24], Coombes et al. [25], and Duff et al. [26] analyzed IGF-1 actions after 4–8 weeks of BC supplementation and observed no significant improvement. In addition, Davison et al. [27] reported no marked changes after the administration of BC supplementation (40 g/day) to athletes for 4 or 12 weeks. On the other hand, a study by Lal et al. [19] indicated that IGF-1 may promote the development of enterocytes and reduce intestinal permeability.

BC contains about 7% of fat, including omega-3 and -6 fatty acids, conjugated linoleic acid, and short-chain fatty acids [28]; especially, the latter may be essential for improving the integrity of the intestinal inner cell membrane [29].

We realize that this is not the first review of BC and its effects on athletes [16,30,31,32,33,34]; however, we intend to analyze and evaluate the published reports on BC supplements and the overall benefits of BC components on intestinal permeability and improvement of athletic performance in athletes. The individual ingredients of BC, viz., lactoferrin, immunoglobulins, fatty acids, and IGF-1, when given alone may not markedly improve the integrity of the intestinal wall thus preventing hyperpermeability; however, the combined administration of BC components appears to produce an additive or synergistic effect in tightening the junctions of the intestinal epithelium by stimulating the secretion of tight-junction proteins (e.g., Claudin-2) and Paneth cells as well as reducing gut inflammation, thereby collectively preventing the seepage of endo-toxins from the lumen into the bloodstream. The involvement of another favorable mechanism, such as BC-induced improvement of the immune system of athletes, also seems an interesting possibility [16]. As alluded to earlier, we will try to answer the following questions in this review:

How does BC supplementation help to reduce gut permeability problems among athletes? 

Which bioactive components of BC have the greatest effectiveness in alleviating gastrointestinal injury? 

What is the mode of action of BC supplementation in influencing gut permeability biomarkers? 

Will the improvement of leaky gut syndrome by BC supplementation also reduce the risk of upper respiratory tract infections in athletes?

## 2. Materials and Methods

### 2.1. Search Strategy

The current study is a systematic review of published literature focusing on the effects of BC supplementation on gut permeability in athletes. This systematic review was conducted following the PRISMA (Preferred reporting items for systematic reviews and meta-analyses) protocol and was registered in PROSPERO, the International Prospective Register of Systematic Reviews, under the registration number CRD42021264064. Four databases were searched: PubMed, Web of Science, Scopus, and EBSCO (Elton Bryson Stephens Company).

The literature search included original papers written in English and published before 15 January 2022. No year restriction was applied. The following keywords or index terms were used: “bovine colostrum,” “leaky gut” or “gut permeability”, “athletes” or “physical effort”, all words in all fields.

### 2.2. Inclusion and Exclusion Criteria

After the database searches, the following inclusion criteria were applied: articles in English language, studies involving males and/or females, adults, studies evaluating physical effort and physical activity, studies involving bovine colostrum supplementation in humans, parameters that measure gut permeability, directly and indirectly, only level 1 OCEBM scale (see Table 1). The following exclusion criteria were adopted: underage subjects, subjects with any disease condition, animal model studies, studies evaluating other parameters than physical effort or exercise, studies involving parameters and markers of gut permeability and bovine colostrum supplements, review papers, meta-analysis.

### 2.3. Data Extraction

**Figure 1:**
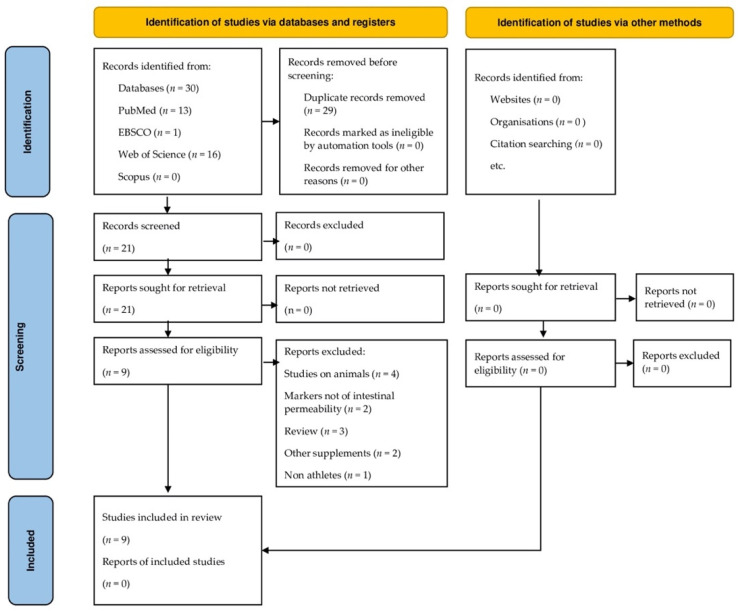
The flowchart figure was drawn according to the statement of the PRISMA protocol [36].

Data were first evaluated by three investigators (H.D., A.K. and M.D.) and then checked independently by two other investigators (A.S.-S. and J.O.-K.). First, all the articles retrieved using the keywords search were downloaded. Then, all replicates were removed, and the article abstracts were analyzed on the basis of the eligibility criteria. Finally, the whole text of articles that met the eligibility criteria (N = 9) was reviewed. Each publication selected for review was critically evaluated. All articles’ full texts were available online. The process is shown in Figure 1.

### 2.4. Quality Assessment

Following the analyses described in Methods, the evidence level was assessed by three independent reviewers (H.D., A.K., and M.D.) using the 2011 method of the Oxford Centre for Evidence-Based Medicine (OCEBM), developed by an International Group of Investigators considering feedback from clinicians, patients, and researchers. The OCEBM method allows the rapid identification of the likely best evidence encouraging clinicians, researchers, and patients autonomously to assess the evidence, as shown in Table 1 [35].

Second, bias analysis was conducted by three investigators (H.D., M.D., and A.K.) using the latest version of the Cochrane Collaboration Risk-of-Bias Tool (Table 2), which is applied in randomized trials [37]. Studies were checked in five domains: bias arising from the randomization process, bias due to deviations from intended innervations, bias due to missing outcome data, bias in the measurement of outcome, and bias in the selection of the reported results. This tool allows the investigator to classify each domain as high risk, of some concern, or low risk. Some concerns were found mainly in the randomization process.

### 2.5. Statistical Analyses

A quantitative representation using a table, without performing further statistics, was obtained. The studies reported data in a different format or/and study design. Hence, it was not possible to extract data for a meta-analysis for statistical comparison. Summary tables were filled with information from each study, including doses of BC, physical activity levels, supplementation period, characteristics of the participants, and outcomes. 

## 3. Results

The literature searches identified 60 potential articles. After the removal of 29 duplicates, 21 records were subjected to article title and abstract screening. The full texts of 21 articles were carefully read, and 9 articles were included in this review. All studies were carried out on male participants, with the exception of one [11]; therefore we could not examine differences between the sexes. 

Table 3 reports nine articles, in which the supplementation period oscillated from 1 to 9 weeks. Permeability after the dual sugar test was increased in one study [38], decreased in four studies [11,39,44,45], remained unchanged in one study [43]. A marker of permeability—intestinal fatty binding protein (I-FABP)—decreased in three studies [39,41,42], while it was unaffected in two studies [40,43].

## 4. Discussion

The analysis of the available literature showed that the BC-induced reduction of intestinal permeability reflects the post-exercise inflammatory response and significantly accelerates the athlete’s recuperation after exercise [1]. BC supplements contain a wide variety of antioxidant and anti-inflammation ingredients, antimicrobials, vitamins and minerals, as well as growth-promoting and immune-boosting compounds that produce beneficial effects in the gastrointestinal tract. Unfortunately, the use of BC in highly trained athletes is problematic because this supplement is included in the WADA anti-doping list (its use is not recommended). Nevertheless, recently published studies have shown highly beneficial effects of BC supplementation in athletes during training sessions [30,31,32]. This systematic review of the published literature intends to highlight the beneficial effects of BC components on intestinal permeability, although the usefulness of IGF-1 present in BC supplementation is debatable.

At first, we tried to answer the question of how BC supplementation influences each biomarker of gut permeability. To begin with, we focused on the two-sugar absorption tests, which are clinically accepted as biomarkers of epithelial integrity and gut permeability [46]. These tests have several limitations related to the lack of reference ranges, standardized methodology, or sugar doses [47]. Despite these limitations, the tests allow the direct assessment of intestinal permeability [34]. In the studies conducted so far, various lactulose, rhamnose, and mannitol combinations have been used. The other factors that interfere with the interpretation of results are the differences in the sugar doses used, because higher doses of sugars may impact on changes in the post-absorption kinetics, which may affect the study results [34]. The results of four studies carried out to investigate the actions of BC supplementation on intestinal permeability showed a reduction [11,39,44,45], while a single study reported an increase [38], and another study found no change [43] in intestinal permeability. The gut permeability alterations found in different studies may be attributed to the BC doses administered (range, 1 g–60 g/day) and the treatment duration. The study with the highest dose (60 g/day) had the longest duration (8 weeks) of BC supplementation, which resulted in increased sugar permeability in the gut [38]. The confounding factors that make the interpretation of the results difficult are if the mega-dose of the supplement was given before or with the meals, whether the highest dose was dissolved in water or milk, and whether the mega-dose could cause indigestion or alter digestibility [38]. However, it is worth mentioning that in the analyzed studies, the participants used different training loads, shown in Table 3, or only the values obtained at rest were analyzed [11,45]. Two research articles showed reduced intestinal permeability after BC supplementation without an exercise test [11,45], which could emphasize that gut permeability did not increase after training loads in those studies. Overall, the BC supplement seems to be a promising therapy to minimize intestinal barrier permeability, especially when measured with the sugar absorption tests. 

Another method used for measuring intestinal permeability is focused on I-FABP (Intestinal fatty acid-binding protein). It has been suggested that I-FABP may be rapidly released into the systemic circulation after pathological damage to the small intestine mucosa [48]. The increase in the plasma level of I-FABP indicates damage to the endothelial cells themselves and not to the tight junctions in the enterocytes. Mainly, changes in I-FABP are related to hypoxia, pH changes, oxidative stress, mechanical stress, and metabolites accumulation in the body [49]. Three studies [39,41,42] revealed decreased levels of I-FABP post-exercise after BC supplementation. Only the work of Morison et al. [43] and McKenne [40] showed no changes in the discussed parameter. In this study, the period of supplementation was only 7 days, and the supplement dose was 1.7 g /kg body wt./day (in the groups of trained persons, the dose was 127 g, and in that of untrained persons, it was 141 g). When taken together, it appears that BC supplementation can reverse intestinal permeability and transfer endotoxins into the systemic circulation. However, further high-quality studies are needed to firmly establish the clinical efficacy and optimal dose levels of BC supplementation.

Due to the limitations of specific diagnostic standards, other biomarkers were used in some studies to analyze the effect of BC on intestinal permeability in physically active people. For example, March et al. [42] observed changes in the level of Bacteroides (total 16S rDNA), suggesting that this biomarker is more sensitive than LPS (lipopolysaccharide) in endotoxemia. Bacteroides constitute approximately 25% of anaerobic bacteria found in the gastrointestinal tract [29]. In the study of March et al. [42], the percentage of Bacteroides significantly decreased after 14 days of BC supplementation (20 g BC/day), indicating a reduction in intestinal permeability and bacterial translocation. This hypothesis was confirmed by the research conducted by Bolke et al. [50], who analyzed the effect of an orally administered BC concentrate (bovine milk preparation—lactobin 56 g/day) in gastrointestinal surgery patients. In the group receiving the supplement, the plasma endotoxin levels were significantly lower than in the control group [50]. It seems that BC supplementation can reduce biomarkers of endotoxemia not only in physically active people but also in clinical patients, which is very promising for future research with BC supplementation. In the future, research should focus on the role of BC in the prevention and treatment of gastrointestinal diseases. Hałasa et al. [45] considered another biomarker called zonulin, and the study sampling was conducted on feces. One of the major mechanisms that regulate intestinal absorption is the production and release of zonulin, a protein that relaxes intestinal tight junctions, allowing paracellular transport through the intestinal mucosa. Zonulin activity allows for paracellular transport in the intestines at the physiological level, while high concentrations of zonulin may indicate pathologically increased intestinal permeability [51]. The zonulin results correlated with the sugar absorption test [52]. The findings of Hałasa et al. [45] showed a reduction in intestinal permeability after BC supplementation in professional athletes. Comparable results were reported by Eslamian et al. in critically ill patients [53]. Therefore, zonulin is another important biomarker that confirmed the decrease of gut permeability after BC supplementation in patients and athletes. 

Next, we tried to answer the question of how BC supplementation reduces gut permeability and restores the integrity of the intestinal endothelium. We would like to mention that the reviewed literature suggests that BC supplementation lowers not only gut permeability but also endotoxemia and has a reparative role in restoring the integrity of the gastrointestinal tract. Enhanced intestinal permeability not only occurs in critically ill patients and in patients with several inflammatory bowel diseases (e.g., IBD, Crohn’s disease) [54,55] but also occurs in athletes and people engaged in strenuous exercises, such as marathon runners [1,4,34]. It seems that dietary supplements based on BC would have beneficial effects on the intestinal permeability in healthy persons as well as in patients with gastrointestinal diseases. It is assumed that the antioxidant and anti-inflammatory compounds present in BC and the modulation of the intestinal microbiota by the active ingredients of BC may be the basis of these actions. Only a few studies showed the indirect effects of dietary ingredients on the composition of the gut microbiota: lactic acid bacteria, bovine colostrum, apple fruit by-products, and essential oils were collectively shown to promote lactic acid strains in the gut [56] that exert health benefits [57]. BC with a *Morinda citrifolia fruit* (Noni) treatment provoked a slight increase in the genus *Akkermansia* [58]. Interestingly, *A. muciniphila* is an intestinal bacterium that has been proposed as a novel health-promoting bacterial species due to its immunomodulatory properties [59]. 

A randomized, double-blind, placebo-controlled clinical trial was conducted in 14 patients using bovine colostrum enemas (100 mL of 10% solution, twice daily) for the treatment of mild to severe distal colitis or inflammatory bowel disease (IBD). The control solution was made from bovine serum albumin. An equivalent volume of the placebo solution enemas was administered b.d. for 4 weeks. Patients in both arms of the trial were also administered mesalazine (1.6 g/day). After 4 weeks of treatment, the histological score markedly improved in five of the eight patients in the BC group, whereas only two of the six patients improved in the placebo group. The results showed that BC enemas can be a novel non-pharmacological therapy for treating IBD, with additional benefits over the use of mesalazine alone. Further studies are needed in a large number of IBD patients to establish the long-term efficacy and safety of BC enemas [60].

It is difficult to answer the question regarding which components present in BC like immunoglobins, lactoferrin, IGF-1, and fat have the greatest potential for sealing the loose junctions of the intestine. BC contains vital nutrients, which in turn can affect overall health and quality of life in athletes. Probably, the health and well-being benefits are derived from the additive/synergetic actions of different ingredients present in the complex mixture of BC. 

Now, the final question: if BC has immune-boosting, antiviral, and antibacterial properties and reduces the gut’s permeability to endotoxins, is this why BC supplementation can lower upper respiratory tract infections? The meta-analysis conducted about the impact of BC on the health and well-being of the athletes so far does not provide unequivocal results. Our systematic review of published studies showed that BC supplementation in athletes lowered the number of days and episodes of upper respiratory tract symptoms (URS) and also lowered the duration of URS episodes [30,32]. Data extracted from another systematic review and meta-analysis [16] suggested that BC supplementation does not change neutrophil, IgA, and IgG concentrations in the blood, as well as salivary IgA. Many authors have proposed that future research is warranted that should focus on the application of more clinically relevant immunological biomarkers to assess the efficacy of BC and determine the optimum doses and duration of BC supplementation in athletes and physically active individuals. Based on our analysis, it appears that BC supplementation has a beneficial role in lowering gut permeability and endotoxemia, and the antimicrobial effects of BC would also help in reducing URS in athletes. The positive impact may derive from the antimicrobial effects of BC on the pathogenic microbial flora in the respiratory tract and lungs. A limited number of studies on athletes have shown that BC supplementation have promising effects on URS [31]. Future research should focus on determining the dose and duration of BC supplementation and include sophisticated biomarkers to assess gut permeability and upper respiratory problems. 

The limitation of this study is the small number of studies were available on the research subjects and the different kind of research protocols were used in these studies, which resulted from the selection according to the protocol.

## 5. Conclusions

A recent review emphasized the role of the biological effects of BC supplementation to improve respiratory health in humans and the potential role of bioactive molecules present in BC as adjunctive therapy for SARS-CoV-2 infection (Coronavirus Disease 2019—COVID-19). There is evidence that BC supplementation is effective against infections and respiratory allergies, as well as in attenuating immunosuppression caused by intense exercise in high-performance athletes [61]. 

Future direction:

• Since bovine colostrum is a food rather than a drug, its adverse effects may be minimal, provided the product is obtained from a reliable source and is prepared using good manufacturing practices (GLP).
• Being a dietary supplement and without any narcotic, steroidal, or euphorigenic properties, it is suggested that BC should be removed from the WADA anti-doping list. While BC products are well tolerated, some patients allergic to dairy products may experience undesirable side effects such as gastrointestinal problems like flatulence and nausea.
• Well-designed, randomized, and placebo-controlled clinical trials are needed to assess the therapeutic potential to alleviate gastrointestinal injury, long-term safety, and efficacy as well as optimal dose levels of colostrum products in athletes and physically active men and women.

## Figures and Tables

**Table 1:** The Oxford 2011 Levels of Evidence [35].

Evidence Level (Treatment Benefits)
Level 1—Systematic review of randomized trials or n-of-1 trials
Level 2—Randomized trial or observational study with dramatic effect
Level 3—Non-randomized controlled cohort/follow-up study
Level 4—Case series, case–control study, or historically controlled study
Level 5—Mechanism-based reasoning

**Table 2:** Cochrane Collaboration Risk-of-Bias Tool [37].

Bucley (2009) [38]	?	+	+	+	+	?
March (2017) [39]	+	+	+	+	+	
McKenne (2017) [40]	?	?	+	+	+	?
Davison (2016) [41]	+	+	+	+	+	
March (2018) [42]	+	+	+	+	+	
Morrison (2014) [43]	?	?	?	+	+	?
Marchbank (2021) [44]	?	+	+	+	+	?
Hałasa (2020) [11]	+	+	+	+	+	
Hałasa (2017) [45]	+	?	+	+	+	?
	Randomisation proecess	Devation from intended intervetion	Missing outcome data	Measurment of the outcome	Selection of the reported results	Overall

Legend: + and green color: Low risk of bias; ? and yellow color: Unclear risk of bias.

**Table 3:** Summary of studies analyzed for gut permeability alterations observed after Bovine Colostrum supplementation.

Autor/Year/OCEMB	Age (y)	N/Sex	Dose	Duration	Sugar Test/Duration of the Urine Collection	I-FABP	Participants	Exercise Test
Bucley et al./2009/1 st level [38]	25+/−4.7	BC group = 9, Whey group = 8, Control group = 13, M	60 g/day	8 weeks	↑, 5 h	-	Regular exercise training for at least three months before the study.	Treadmill running test until the participant reached volitional exhaustion.
March et al./2017/1 st level [39]	26+/−5	18, M	20 g/day	14 weeks	↓, 5 h	↓	All regular exercisers. VO_2_ peak 56.3 +/−6.5 mL·kg/min	20 min run at a constant speed equivalent to 80% VO_2_ peak on treadmill, 1 % grade, 22.1+/−1.7 C, 37+/−8% humidity.
McKenne et al./2017/1 st level [40]	20+/−2	10, M	20 g/day, two doses daily 10 g each	14 weeks	-	←	VO_2_ max 55.8 +/−3.79 mL·kg/min, variable running time in two conditions CON to BC	Participants ran for 46 ± 7.75 min at 40 °C and 50% RH using a temperature- and humidity-controlled environmental chamber. Exercise was terminated after 60 min, if Tcore exceeded 40 °C, heart rate rose above 195 bpm, or if participants asked to terminate the trial.
Davison et al./2016/1 st level [41]	Mean 25	8, M	20 g bovine colostrum, 10 g BC capsule, each were taken 2 times	14 days	-	↓	Active individuals who regularly exercised 4 times/week VO_2_ max 59.6 +/−1.8 mL·kg/min	Treadmill running for 20 min at 80% of the VO_2_ max.
March et al./2018/1 st level [42]	26+/−6	12, M	20 g/day, 10 g in the morning, and the same with the evening meal	14 days	-	↓	VO_2_ peak 55.8 +/−4.8 mL·kg/min	Constant speed equivalent to 70% VO_2_ peak treadmill with a 1% grade for 60 min or until core temperature reached 40 °C. Climatic chamber was maintained at 30.0 ± 0.1 °C, and 60 ± 0% relative humidity.
Morrison et al./2014/1 st level [43]	23+/−4, 21+/−2	*n* = 7 trained group (TG), *n* = 8 untrained group (UG), M	1.7 g/kgmc/day	7 days	←, 5 h	←	TG 60 mL·kg^−1^·min^−1^ and trained at least 6 times per week for at least 60 min per training session, UG less than 50 mL·kg^−1^·min^−1^ and participated in physical activity less than 3 times per week	Exercise consisted of 15 min cycling at a fixed load, initially eliciting 50% HRR (cycle 1), running for 30 min at a fixed speed initially eliciting 80% HRR (run 1), 30 min running maximal-distance trial (run 2), 15 min cycle. Environmental chamber (30 °C, 50% RH) with graded airflow based on the participant’s running speed (wind speed: 3.5 m·s^−1^ to 4.5 m·s^−1^).
Marchbank et al./2021/1 st level [44]	Mean 26	12, M	20 g/day	14 days	↓, 5 h	-	All subjects were regular exercisers and took part in running as part of their training; seven were runners, two participated in boxing, and three participated in rugby. VO_2_ max 53.3 +/−6.8 mL·kg/min	20 min run at a constant speed equivalent to 80% VO_2_ peak on treadmill, 1 % grade, 22.1+/−1.7 C, 37+/−8% humidity
Hałasa et al./2020/1 st level [11]	Mean 34.5	*n* = 7, *n* = 8, *n* = 9, *n* = 9, M, F	500 mg 2/day harvested in 2 h	20 days	↓, 6 h	-	A group of 36 healthy volunteers, 30 males and 6 females, active athletes from various sport disciplines, including mixed martial arts (10), triathlon (11), cycling (9), water polo (6).	No exercise test.
Hałasa et al./2017/1 st level [45]	Mean 27.5	*n* = 7, *n* = 7, M	500 mg 2/day in the morning and in the evening, m 30 min before meal	20 days	↓, 6 h	-	Martial arts fitters in active training at the middle of the competition season.	No exercise test.

Symbols/Abbreviations used: ←—unchanged; ↑—increased; ↓—decreased; BC—bovine colostrum; HR—heart rate; HRR—heart rate reserve; TC—trained group; Tcore—core temperature; UG—untrained group; VO_2_ peak—the highest/maximum oxygen consumption achieved during a clinical/research graded exercise test; VO_2_ max—the maximal aerobic power defined as the maximum amount of oxygen that an individual can utilize during intense or maximal exercise, M—Male, F—female.

## Data Availability

Not applicable.
